# Supplementary material for: Illusory sound texture reveals multi-second statistical completion in auditory scene analysis
Source: Nat Commun. 2019 Nov 8;10:5096. doi: 10.1038/s41467-019-12893-0 (PMC6841952; doi:10.1038/s41467-019-12893-0)
Supplement: Supplementary file 1 — Supplementary Information [file 41467_2019_12893_MOESM1_ESM.pdf]

Supplementary Information

Illusory sound texture reveals multi-second statistical completion in auditory scene analysis

Richard McWalter[1,2,3] and Josh H. McDermott[1,2,3,4]

1. Department of Brain and Cognitive Sciences, MIT, Cambridge MA 02139
2. Center for Brains, Minds and Machines, MIT, Cambridge MA 02139
3. McGovern Institute for Brain Research, MIT, Cambridge MA 02139
4. Program in Speech and Hearing Biosciences and Technology, Harvard University, Cambridge MA 02138

Inducer	Masking noise SNR (dB)	Inducer	Masking noise SNR (dB)
Bees	-18*	Pneumatic drill	-14
Birds in spring	-16.5	Pneumatic rills (road-works)	-14
Cat lapping milk	-14.5	Pouring coffee beans	-17.5
Frogs	-12.5	Pouring coins	-16
Swamp insects	-16	Static	-16
Rattlesnake	-16.5	Scratching beard	-17
Sparrows (large excited group)	-16.5	Shaking paper	-15
Summer day in the south	-12.5	Ship anchor up	-13.5
Swamp noises	-16.5	Snare steel sequence	-23
Swarm of bees	-17.5	Radio static	-12.5
Fire	-18	Steam train slow	-20
Heavy rain on hard surface	-17	Tambourine shaking	-14
Wind whistling	-17.5	Tapping rhythm	-16
Rain beating against window	-13.5	Train	-19
Rain	-14.5	Windshield wipers	-20
AC unit	-23	Animated crowd in a large hall	-13.5
AM radio noise	-14	Rhythmic applause	-19
Blender	-16.5	Applause in large hall	-18
Brushing teeth	-15	Applause	-15
Car interior	-17.5	Babble	-18
Church bells	-15.5	Enthusiastic applause	-17.5
City room teletype	-16	German rail station announcer	-21.5
Coin rolling on plate	-14.5	Large railway station	-15.5
Crumpling paper	-17.5	Men marching	-17
Crunching cellophane	-14	Music (apache)	-21.5
Dinner triangle	-17.5	Music (bluegrass)	-20.5
Drumroll long	-19	Music (orchestra)	-21.5
Lawn edger	-15.5	Speech (German female)	-23.5
Electric drill	-16.5	Speech (German male)	-23
Firecrackers	-17	Bathroom sink	-15.5
Frying bacon	-14.5	Bubbles	-23
Galloping horse	-24	Bubbling water	-19
Hammering copper	-18	Cave drop	-20.5
Hand sanding	-12.5	Fast running river	-20
Helicopter	-21.5	Ocean wave slow	-21.5
Jingling coins	-16	River running over shallows	-18
Jogging on gravel	-12.5	Stream small	-19
Manual typewriter	-14	Water dribbling	-20
Modulated radio static	-14.5	Water lapping gently	-19.5
Newspaper printing press	-11.5	Water movement	-21

* Negative SNR value corresponds to the noise (masker) being higher in level than the signal (inducer).

Supplementary Table 1 Sounds and masking noise levels used in Experiment 1a. Masking noise SNR (dB) indicates the relative difference in amplitude between the inducer sound and the Gaussian masking noise, selected by the first author to produce masking of the texture by the noise when they were superimposed.

Texture Inducer	Masking noise SNR (dB)	Environmental Inducer	Masking noise SNR (dB)
Car interior	-17.5	Cat lapping milk	-14.5
Lawn edger	-15.5	Church bells	-15.5
Pouring coffee beans	-14.5	Crumpling paper	-17.5
Animated crowd in hall	-13.5	Pouring coins	-16
Applause in large hall	-18	Bubbles	-23
River running over shallows	-18	Water movement	-21
**Speech / Music Inducer**	**Masking noise SNR (dB)**	**Periodic Inducer**	**Masking noise SNR (dB)**
Speech (German female 1)	-23.5	Drum sample (2)	-20
Music (Bluegrass)	-20.5	Ticking clock	-19
Music (Orchestral)	-21.5	Galloping horse (3)	-16.5
German rail station announcer	-21.5	Marching men	-14
Speech (German male)	-23	Sawing wood	-15.5
Speech (English female)	-21	Hammering copper	-18

Supplementary Table 2 Sounds and masking noise levels used in each condition of Experiment 2 (varying masker duration).

Inducer	Masking noise SNR (dB)	Inducer	Masking noise SNR (dB)
Swamp insects	-16	Rain beating against window	-13.5
Car interior	-17.5	Firecrackers	-17
Jogging on gravel	-12.5	Shaking paper	-15
Applause in large hall	-18	German rail station announcer	-21.5
Music (Bluegrass)	-20.5	Bubbling water	-19
Rattlesnake	-16.5	Brushing teeth	-15
Lawn edger	-15.5	Helicopter	-21.5
Pouring coffee beans	-17.5	Tapping rhythm	-16
Babble	-18	Large railway station	-15.5
Speech (German male)	-23	Water movement	-21

Supplementary Table 3 Sounds and masking noise levels used in Experiment 3 (extent estimation task).

Inducer	Masking noise SNR (dB)	Inducer	Masking noise SNR (dB)
Rattlesnake	-16.5 (-6.5)	Pneumatic drill	-14 (-2.5)
Heavy rain on a hard surface	-17 (-4.5)	Pneumatic drills (road works)	-14 (-4.5)
Wind whistling	-17.5 (-10.5)	Pouring coffee beans	-17.5 (-16.5)
Rain	-14.5 (-6.5)	Ship anchor	-13.5 (-3.5)
Blender	-16.5 (-9.5)	Radio static	-12.5 (-4)
Car interior	-17.5 (-11.5)	Babble (large hall)	-18 (-8.5)
Crunching cellophane	-14 (-7)	Applause in large hall	-18 (-5.5)
Lawn edger	-15.5 (-9.5)	Applause	-15 (-6.5)
Electric drill	-16.5 (-8)	Fast running river	-20 (-3.5)
Frying bacon	-14.5 (-8.5)	River running over shallows	-18 (-7.5)

Supplementary Table 4 Sounds and masking noise levels used in Experiment 5 (effect of gaps). These sounds were also used in Experiments 4a and 4b. Masking Noise SNR was that used in Experiment 5. The values in parentheses are the masking thresholds measured in Experiment 4a. The noise levels used in Experiment 5 were higher than these thresholds (lower SNR), which is conservative with respect to ensuring that the noise could have masked the inducer.

Reference	Masking Noise SNR (dB)
River running over shallows	-18
Pneumatic drill	-14
Applause	-16
Lawn edger	-17.5

Supplementary Table 5 Sounds and masking noise levels used in Experiment 6 (texture step experiment). The listed masking noise SNR was used for all stimuli for each reference texture.

Texture	Non-texture	Masking noise SNR (dB)
Air conditioning unit	Coin rolling on a plate	-14.5
Car interior	Crumpling paper	-17.5
Wind whistling	Dinner triangle	-17.5
Lawn edger	Firecrackers	-17
Applause in large hall	Galloping horse	-24
Electric drill	Hammering copper	-18
Pneumatic drill	Jingling coins	-16
Fast running river	Jogging on gravel	-12.5
Animated crowd in a large hall	Snare steel sequence	-23
Pouring coffee beans	Tapping rhythm	-17.5
Kitchen blender	Speech (German, male)	-23
River running over shallows	Music (bluegrass)	-20.5
Rain	Men marching	-17
Applause	Tambourine shaking	-14
Large railway station	Music (orchestra)	-21.5
Crunching cellophane	Shaking paper	-15
Heavy rain on a hard surface	Cat lapping milk	-14.5
Frying bacon	Water dribbling	-20
Rattlesnake	Water movement	-21
Pneumatic drills at road works	Birds in spring	-16.5

Supplementary Table 6 Sounds and masking noise levels used in Experiment 7 (texture continuity with concurrent non-textures).

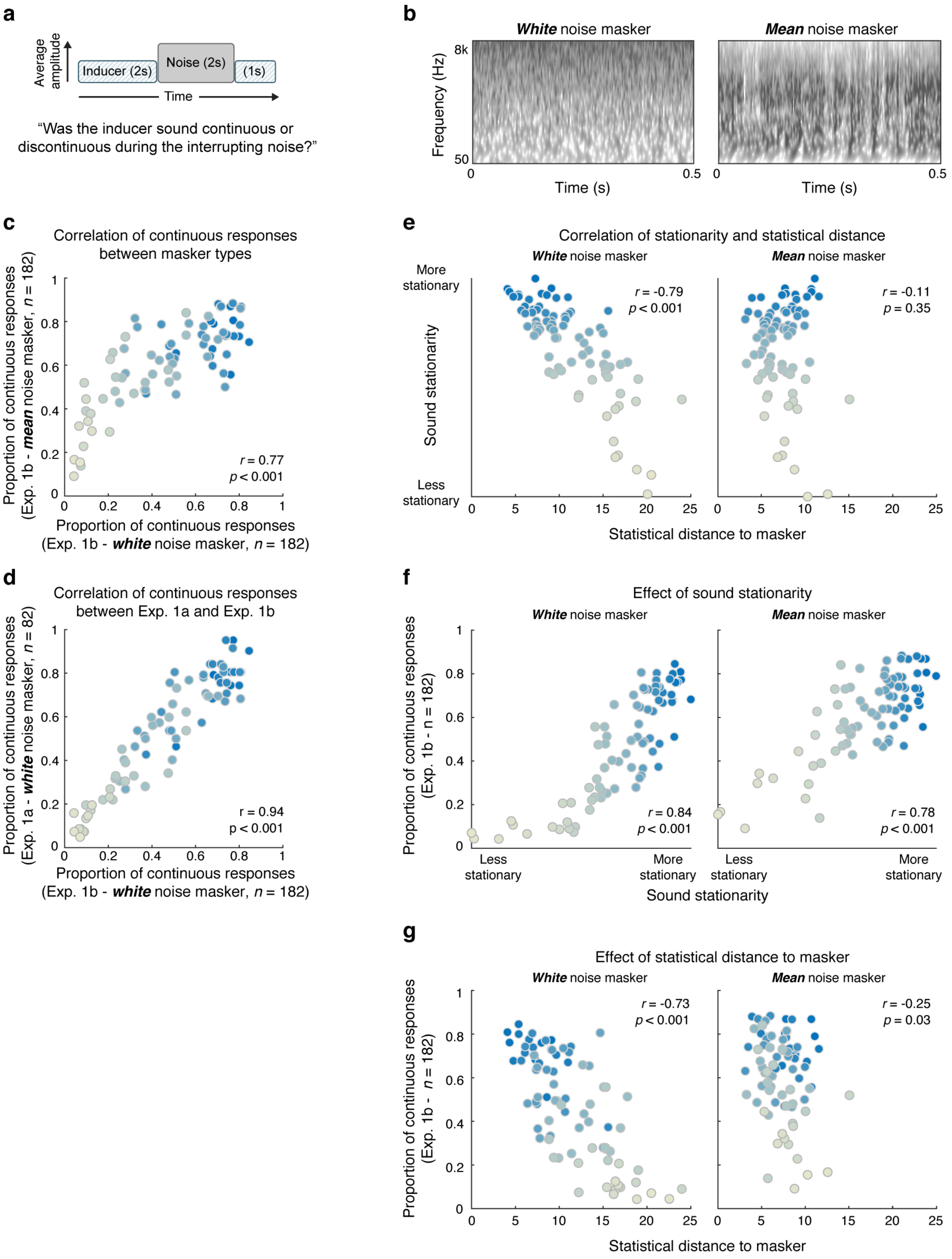

Supplementary Fig. 1 Results of Experiment 1b. **a** Schematic of stimulus and task. **b** Cochleagrams of excerpts of white noise masker (left) and mean noise masker (right). **c** Correlation between experimental results (proportion of trials on which the inducer was judged to be present during the noise) for white noise masker and mean noise masker in Experiment 1b. Each data-point represents an inducer sound used in Experiment 1b. Here and elsewhere, dot color corresponds to the statistical stationarity measure from Figure 2f. Here and elsewhere, *r* values give Pearson correlation. Continuity judgments were correlated across masker types, but higher overall for the mean masker. **d** Correlation between perceptual continuity judgments for white noise masker in Experiment 1a and Experiment 1b (which differed in the participants, as well as in the presence of the mean noise masker condition). Results were similar across experiments. **e** Correlation of statistical distance to the masker and sound stationarity, for white noise masker (left) and mean noise masker (right) used in Experiment 1b. **f** Correlation between perceptual continuity judgments and sound stationarity, for white noise masker (left) and mean noise masker (right) conditions. **g** Correlation between perceptual continuity judgments and statistical distance to the masker, for white noise masker (left) and mean noise masker (right) conditions.

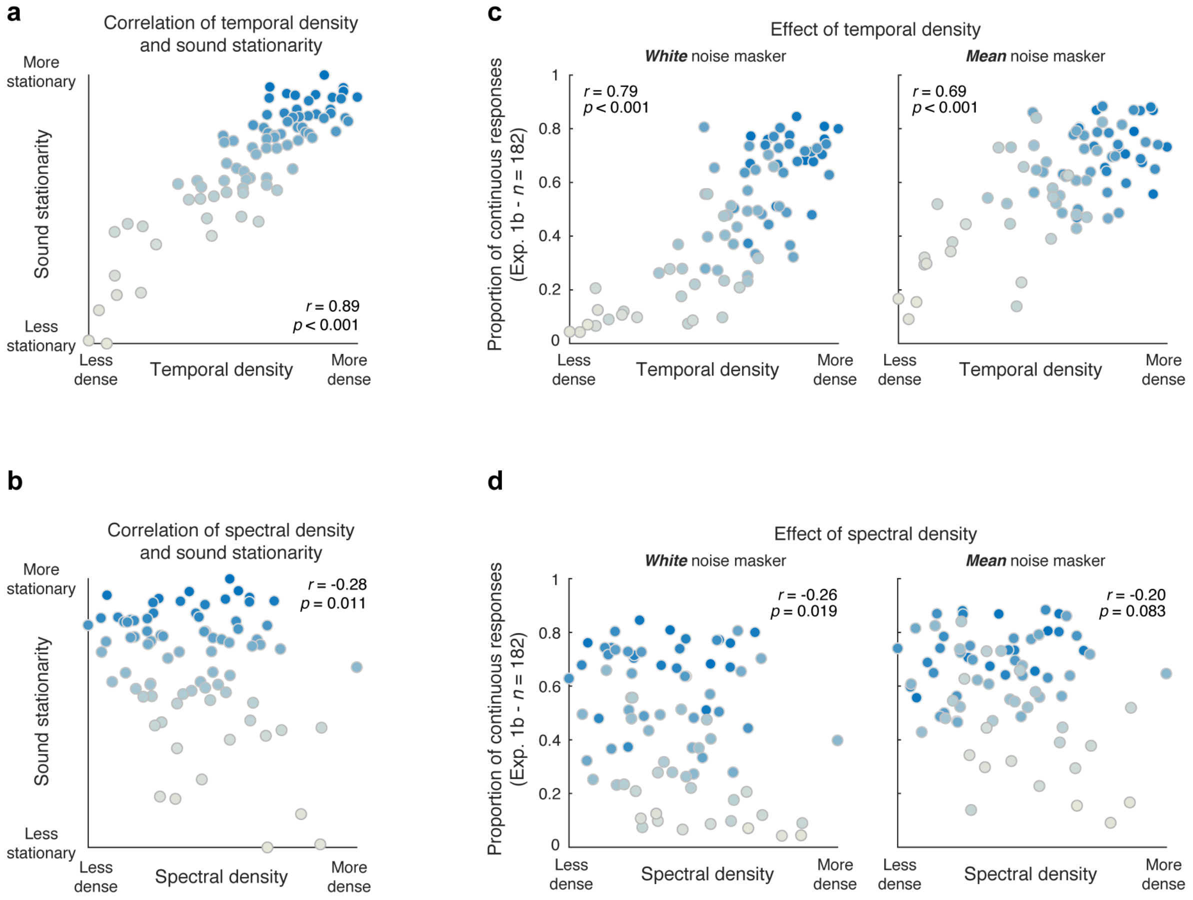

Supplementary Fig. 2 Temporal and spectral density analyses of Experiment 1b. **a** Correlation between temporal density and sound stationarity measures. Here and elsewhere, *r* values give Pearson correlation. Data-point color code corresponds to sound stationarity measure from Figure 2f. **b** Correlation between spectral density and sound stationarity measure. **c** Correlation between temporal density of inducer sound and perceptual continuity, for white noise masker (left) and mean noise masker (right) conditions of Experiment 1b. **d** Correlation between spectral density of inducer sound and perceptual continuity, for white noise masker (left) and mean noise masker (right) conditions of Experiment 1b.

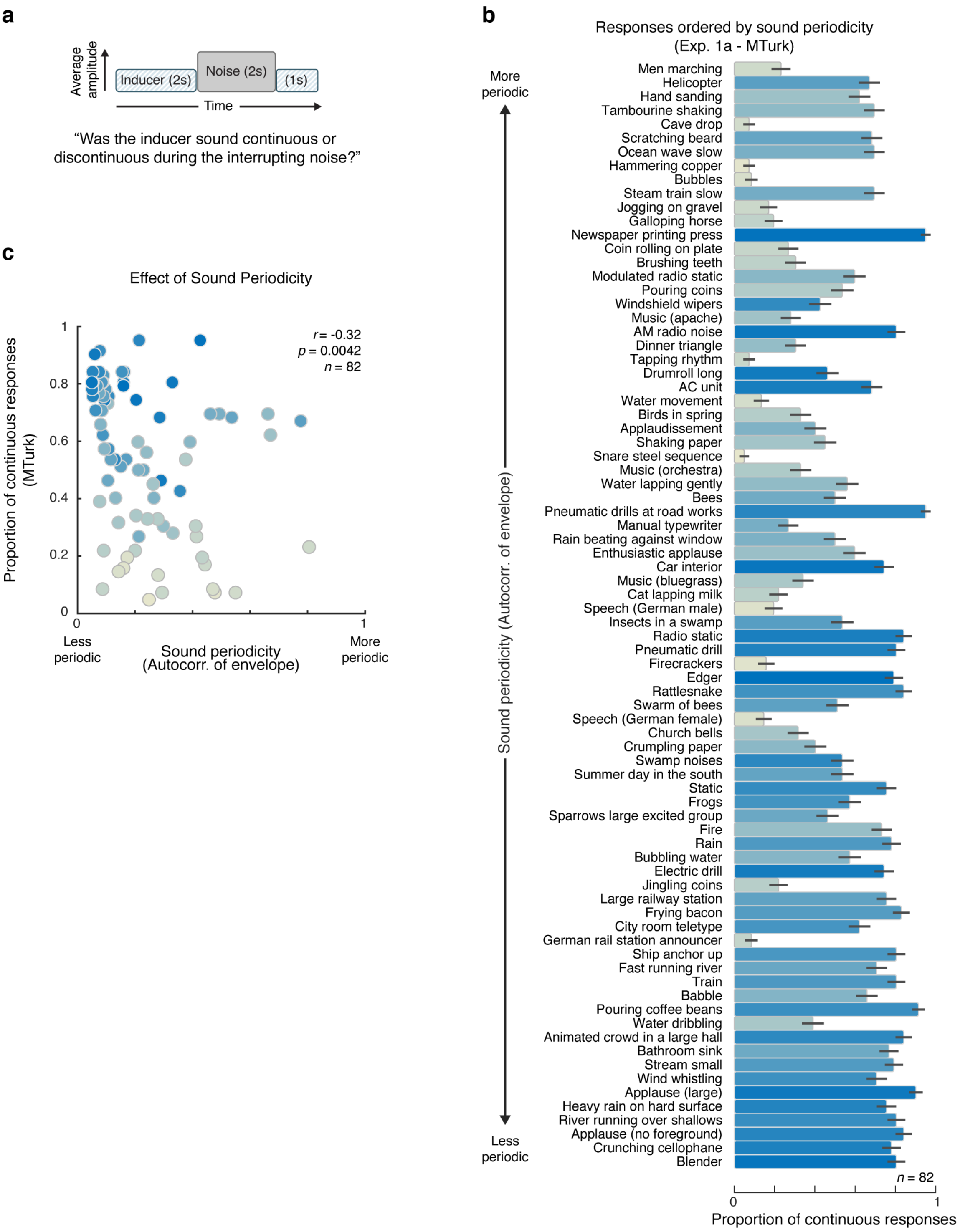

Supplementary Fig. 3 Results of Experiment 1a ordered by sound periodicity. **a** Schematic of stimulus and task for Experiment 1. **b** Results of Experiment 1a. Analogous to Figure 2f except that sounds are sorted by their periodicity. Periodicity was computed from the autocorrelation of the Hilbert envelope of the sound waveform, downsampled to 400 Hz. The periodicity measure was the height of the largest autocorrelation peak for lags between 0.125 s and 0.5 s, normalized by the autocorrelation at lag 0. Error bars show SEM. Color corresponds to the stationarity measure shown in Figure 2f. **c** Mean proportion of continuous responses plotted as a function of sound periodicity. *r* value is Pearson correlation.

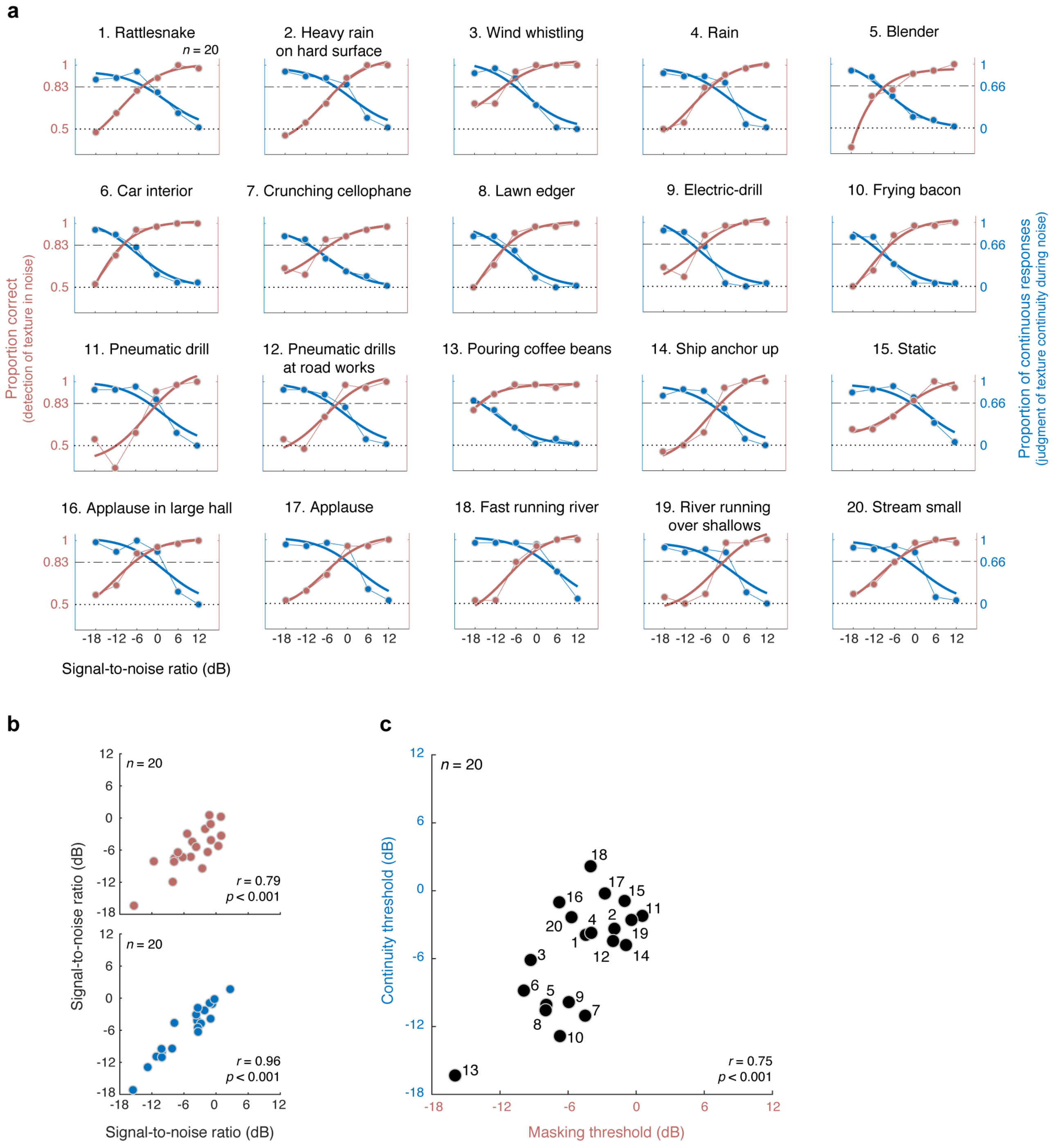

Supplementary Fig. 4 Results for the masking and continuity experiments (4a/4b) plotted separately for individual sounds. To increase power, this analysis combined the data from Experiments 4a/4b with that of a pilot experiment (n = 10) that was identical except that the stimuli for a given reference sound were generated from a single synthetic texture exemplar (yielding n = 20 in total). **a** Masking and continuity curves for individual sounds. The data points and light/thin lines show the mean response across SNR values. The dark/thick lines show logistic function fits. The horizontal dashed line shows the threshold value (masking 0.833, continuity 0.666) used to relate masking and continuity in b. **b** Reliability of masking and continuity threshold measurements. Subpanels show test-retest reliabilities for the continuity experiment (top) and masking experiment (bottom). Reported Pearson correlation is the average of 10,000 test-retest splits (each participant performed two trials per condition, which were randomly assigned to a split); graphs show results from one example split. **c** Continuity thresholds plotted against masking thresholds, for individual sounds. The r value is the Pearson correlation between masking and continuity thresholds, corrected for attenuation. Index refers to sounds in subplot a. Masking and continuity thresholds were generally similar, and co-varied across sounds to some extent.

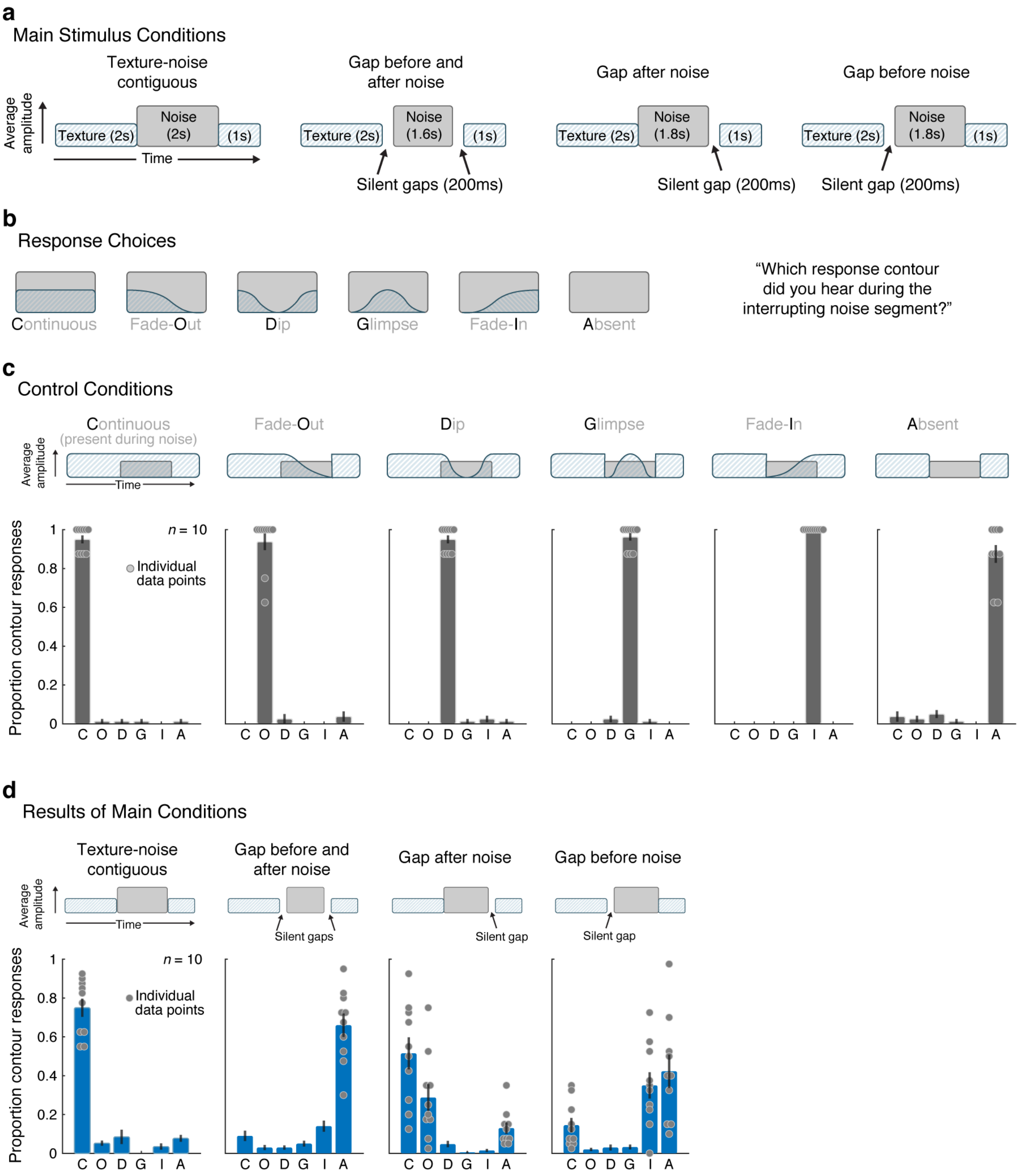

Supplementary Fig. 5 Replication of Experiment 5 with real-world sound texture recordings. (caption duplicated from Figure 6 caption)
a Listeners heard a synthetic inducer texture interrupted with masking noise and reported their perceptual experience during the interrupting noise segment. The experiment included 4 conditions, differing in the contiguity of the texture and noise (via silent gaps inserted before and/or after the noise). See Supplementary Figure 3 for analogous experiment with real-world texture recordings (which yielded similar results).
b Listeners chose one of six response contours to describe their perceptual experience during the interrupting noise segment. The contour response code is indicated as the bolded letter for each response (e.g. "C" for "**C**ontinuous"). **c** To confirm task comprehension/compliance, the experiment included control trials where the texture was physically present during the intermediate noise segment and amplitude modulated according to one of the response contours. The stimulus for each condition is schematized above each of the six subplots. Graphs plot the proportion of trials on which each response was chosen. Data for individual participants is plotted as dots for the response choices selected above chance levels for each condition. **d** Results of main experimental conditions. Each subplot corresponds to a condition (shown schematically above). Data for individual participants is plotted as dots for the response choices selected above chance levels for each condition. Error bars plot SEM.

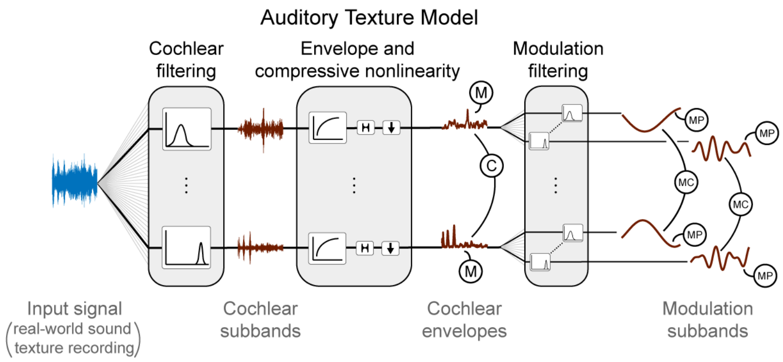

Supplementary Fig. 6 Auditory texture model. The model was adapted from that of McDermott and Simoncelli (2011). Statistics are measured from an auditory model capturing the tuning properties of three stages of the peripheral and subcortical auditory system. The cochlear envelope marginal statistics (M) comprise the mean, coefficient of variance, and skewness. Pair-wise envelope (C) correlations were computed between neighboring cochlear envelope bands. The modulation subband statistics comprise the modulation power (MP; the variance of the modulation normalized by the corresponding total cochlear envelope variance) and modulation correlations (MC) between modulation subbands.

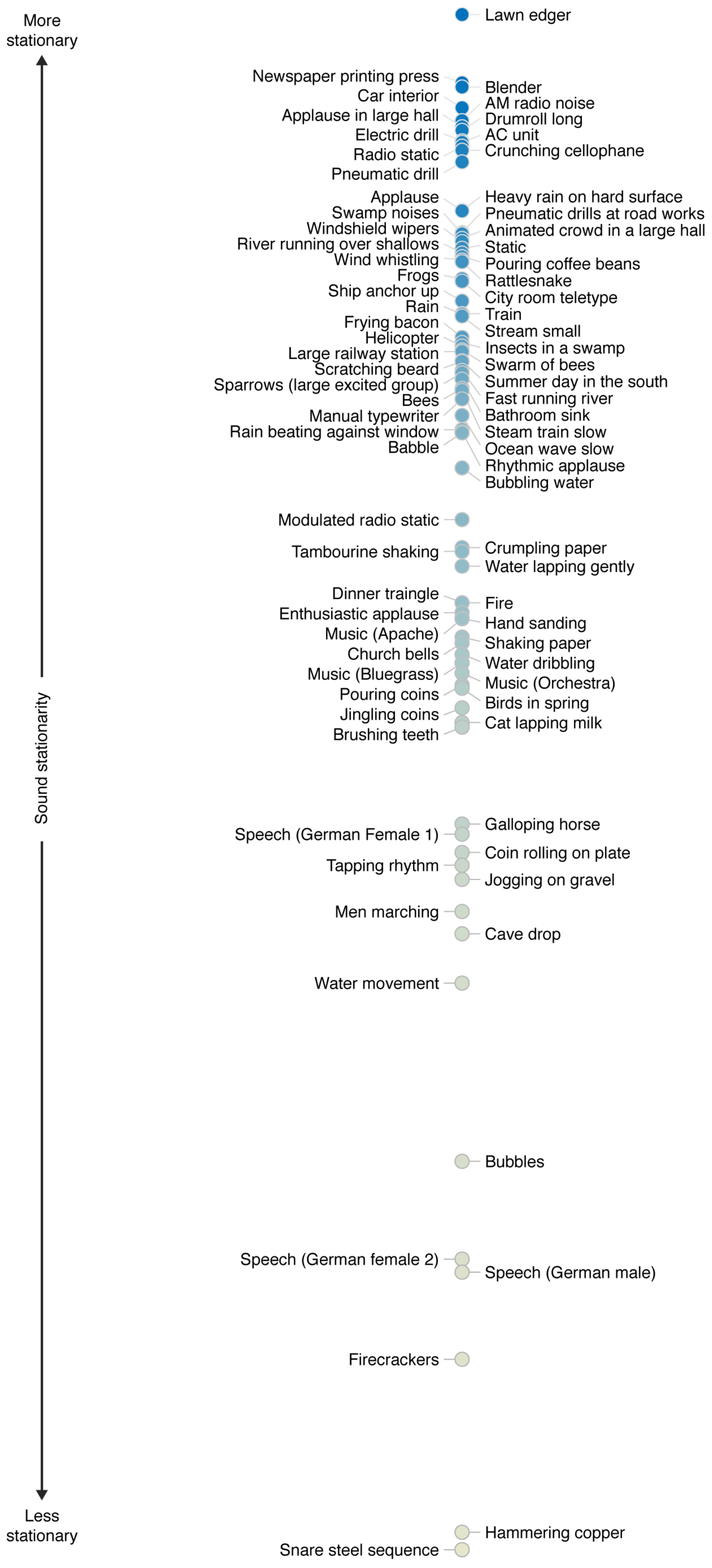

Supplementary Fig. 7 Stationarity of sounds used in Experiment 1 (80 sounds).

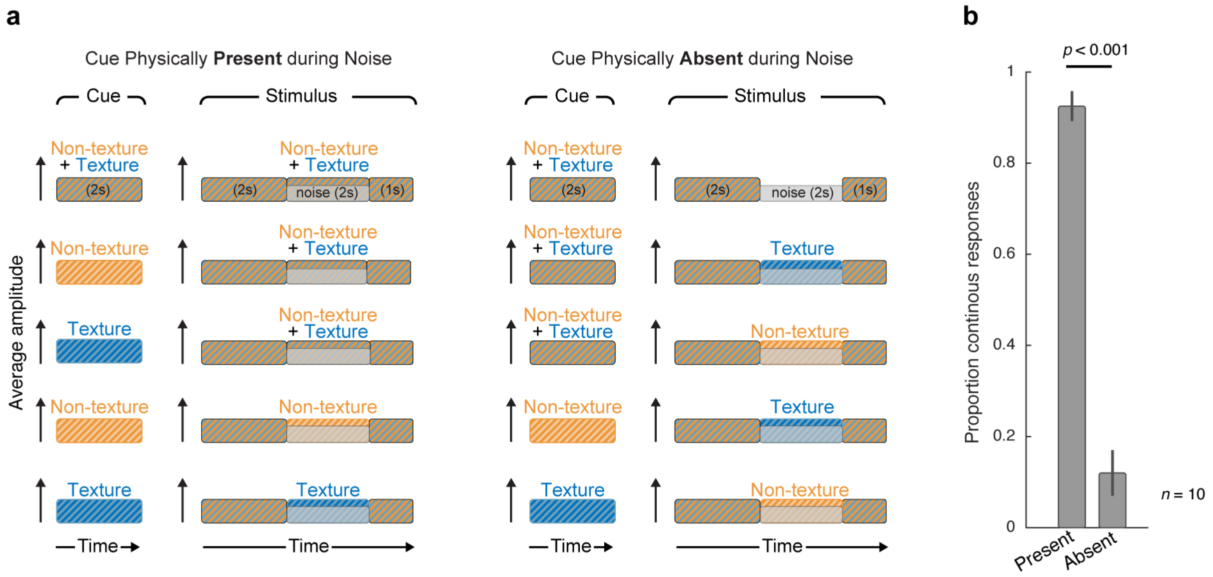

Supplementary Fig. 8 Control conditions for Experiment 7. **a** The stimuli varied in the sounds that were physically present during the noise. In five of the conditions the cued sound was physically present and in the other five the cued sound was physically absent during the noise segment. **b** Results for control conditions shown in a. Proportion of trials on which participants judged the cued stimulus to continue during the noise, averaged within the two groups of conditions (present or absent). The results indicate that participants were performing the task as intended, in that they reported perceptual continuity when the cued sound was physically continuous, but not when it was unambiguously absent. P value is from a two-tailed paired *t* test comparing continuous responses between cue present and absent conditions. Error bars plot SEM.
