# Supplementary material for: Illusory sound texture reveals multi-second statistical completion in auditory scene analysis
Source: Nat Commun. 2019 Nov 8;10:5096. doi: 10.1038/s41467-019-12893-0 (PMC6841952; doi:10.1038/s41467-019-12893-0)
Supplement: Supplementary file 3 — Reporting Summary [file 41467_2019_12893_MOESM3_ESM.pdf]

nature research

Corresponding author(s): Richard McWalter, Josh McDermott

Last updated by author(s): Aug 27, 2019

Reporting Summary

Nature Research wishes to improve the reproducibility of the work that we publish. This form provides structure for consistency and transparency in reporting. For further information on Nature Research policies, see Authors & Referees and the Editorial Policy Checklist.

Statistics

For all statistical analyses, confirm that the following items are present in the figure legend, table legend, main text, or Methods section.

n/a	Confirmed	
☐	☒	The exact sample size (*n*) for each experimental group/condition, given as a discrete number and unit of measurement
☐	☒	A statement on whether measurements were taken from distinct samples or whether the same sample was measured repeatedly
☐	☒	The statistical test(s) used AND whether they are one- or two-sided<br>*Only common tests should be described solely by name; describe more complex techniques in the Methods section.*
☒	☐	A description of all covariates tested
☐	☒	A description of any assumptions or corrections, such as tests of normality and adjustment for multiple comparisons
☐	☒	A full description of the statistical parameters including central tendency (e.g. means) or other basic estimates (e.g. regression coefficient) AND variation (e.g. standard deviation) or associated estimates of uncertainty (e.g. confidence intervals)
☐	☒	For null hypothesis testing, the test statistic (e.g. *F*, *t*, *r*) with confidence intervals, effect sizes, degrees of freedom and *P* value noted<br>*Give P values as exact values whenever suitable.*
☒	☐	For Bayesian analysis, information on the choice of priors and Markov chain Monte Carlo settings
☒	☐	For hierarchical and complex designs, identification of the appropriate level for tests and full reporting of outcomes
☐	☒	Estimates of effect sizes (e.g. Cohen's *d*, Pearson's *r*), indicating how they were calculated

Our web collection on statistics for biologists contains articles on many of the points above.

Software and code

Policy information about availability of computer code

Data collection	In-lab experiments were implemented using Mathworks MATLAB v2016a (or later) and PsychToolBox v3. Online experiments were designed using HTML and javascript and run on Amazon's MechanicalTurk crowdsourcing platform.
Data analysis	Data analysis was conducted with Mathworks MATLAB v2016a (or later) using in-built functions and custom scripts/functions.

For manuscripts utilizing custom algorithms or software that are central to the research but not yet described in published literature, software must be made available to editors/reviewers. We strongly encourage code deposition in a community repository (e.g. GitHub). See the Nature Research guidelines for submitting code & software for further information.

Data

Policy information about availability of data

All manuscripts must include a data availability statement. This statement should provide the following information, where applicable:

- Accession codes, unique identifiers, or web links for publicly available datasets
- A list of figures that have associated raw data
- A description of any restrictions on data availability

Data available upon request.

Field-specific reporting

Please select the one below that is the best fit for your research. If you are not sure, read the appropriate sections before making your selection.

☐ Life sciences ☒ Behavioural & social sciences ☐ Ecological, evolutionary & environmental sciences

Behavioural & social sciences study design

All studies must disclose on these points even when the disclosure is negative.

Study description	The experiments involved a variety of psychophysical tasks in which participants heard sounds and made judgments about them. Data were quantitative.
Research sample	In-lab participants were recruited from the local community. The majority of in-lab participants were undergraduate students from the Boston area. Online experiments were conducted using the MechanicalTurk workforce, Geo-restricted to North America.
Sampling strategy	Pilot studies were conducted to estimate sample sizes required to achieve reliability r > 0.9 (see Methods).
Data collection	In-lab experiments were conducted using computers. Online experiments were conducted on the Amazon MechanicalTurk platform on web enabled devices. Online participants were screened with a headphone check (Woods et al. 2017) to help ensure good listening conditions.
Timing	September 2018 - August 2019
Data exclusions	All in-lab participants were included in data analysis except for Experiment 6, where participants were screened for task compliance based on their overall performance on the discrimination task (13/16 passed exclusion criteria). Online participants were required to complete a "headphone check" and complete training trials with performance exceeding 75% before continuing to the main experiment. All data collected from the main online experiments were used in the analysis.
Non-participation	none.
Randomization	*If participants were not allocated into experimental groups, state so OR describe how participants were allocated to groups, and if allocation was not random, describe how covariates were controlled.*

Reporting for specific materials, systems and methods

We require information from authors about some types of materials, experimental systems and methods used in many studies. Here, indicate whether each material, system or method listed is relevant to your study. If you are not sure if a list item applies to your research, read the appropriate section before selecting a response.

Materials & experimental systems

n/a	Involved in the study	
☒	☐	Antibodies
☒	☐	Eukaryotic cell lines
☒	☐	Palaeontology
☒	☐	Animals and other organisms
☐	☒	Human research participants
☒	☐	Clinical data

Methods

n/a	Involved in the study	
☒	☐	ChIP-seq
☒	☐	Flow cytometry
☒	☐	MRI-based neuroimaging

Human research participants

Policy information about studies involving human research participants

Population characteristics	see above
Recruitment	In-lab participants were recruited from a mailing list used by our department for this purpose (targeting local residents of Boston) and word of mouth. The majority of in-lab participants were undergraduate students from th Boston area. Online experiments were conducted using the MechanicalTurk workforce, Geo-restricted to North America.
Ethics oversight	Massachusetts Institute of Technology Committee on the Use of Humans as Experimental Subjects (COUHES) approved all experiments.

Note that full information on the approval of the study protocol must also be provided in the manuscript.
